# Women’s views and experiences of two alternative consent pathways for participation in a preterm intrapartum trial: a qualitative study

**DOI:** 10.1186/s13063-017-2149-3

**Published:** 2017-09-09

**Authors:** Alexandra Sawyer, Celine Chhoa, Susan Ayers, Angela Pushpa-Rajah, Lelia Duley

**Affiliations:** 1 Centre for Health Research, School of Health Sciences, University of Brighton, Falmer, BN1 9PH UK; 2 Centre for Maternal and Child Health Research, School of Health Sciences, City University London, London, EC1R 1UW UK; 3 Department of Dermatology, Guy’s Hospital, London, SE1 9RT UK; 4 Nottingham Clinical Trials Unit, University of Nottingham, Nottingham, NG7 2UH UK

**Keywords:** Clinical trials, Ethics, Consent, Oral assent, Preterm birth

## Abstract

**Background:**

The Cord Pilot Trial compared alternative policies for timing of cord clamping at very preterm birth at eight UK hospitals. In addition to standard written consent, an oral assent pathway was developed for use when birth was imminent. The aim of this study was to explore women’s views and experiences of two alternative consent pathways to participate in the Cord Pilot Trial.

**Methods:**

We conducted a qualitative study using semi-structured interviews. A total of 179 participants in the Cord Pilot Trial were sent a postal invitation to take part in interviews. Women who agreed were interviewed in person or by telephone to explore their experiences of two consent pathways for a preterm intrapartum trial. Data were analysed using inductive systematic thematic analysis.

**Results:**

Twenty-three women who gave either written consent (*n* = 18) or oral assent followed by written consent (*n* = 5) to participate in the trial were interviewed. Five themes were identified: (1) understanding of the implications of randomisation, (2) importance of staff offering participation, (3) information about the trial and time to consider participation, (4) trial secondary in women’s minds and (5) reasons for agreeing to take part in the trial. Experiences were similar for the two consent pathways. Women recruited by the oral assent pathway reported being given less information about the trial but felt it was sufficient to make a decision regarding participation. There were gaps in women’s understanding of the trial and intervention, regardless of the consent pathway.

**Conclusions:**

Overall, women were positive about their experiences of being invited to participate in the trial. The oral assent pathway seems an acceptable option for women if the intervention is low-risk and time is limited.

**Trial registration:**

ISRCTN Registry, ISRCTN21456601. Registered on 28 February 2013.

**Electronic supplementary material:**

The online version of this article (doi:10.1186/s13063-017-2149-3) contains supplementary material, which is available to authorized users.

## Background

High-quality randomised trials are the gold standard for evaluating interventions to improve outcomes for babies born very preterm (<32 weeks of gestation), who are at increased risk for neonatal complications and neurodevelopmental problems [1, 2]. The Cord Pilot Trial investigators compared alternative policies for timing of cord clamping at very preterm birth at eight UK hospitals [3, 4]. Very preterm birth can be rapid and unexpected and is often a difficult and stressful time for women and their partners, so approaching women to offer participation in a randomised trial may be difficult. Yet, these women often give birth to babies at high risk of poor outcome; hence, it was particularly important that they were offered the opportunity to be included in the trial.

In addition to the usual procedure for written consent, we therefore developed a two-stage ‘oral assent’ pathway for consent for use when birth was imminent [4]. This oral assent pathway was developed in partnership with parent representatives from Bliss, the special care baby charity, and the National Childbirth Trust, and it is in line with guidance on valid consent for research while in labour [5]. The aim of this study was to describe women’s views and experiences of the two alternative consent pathways for participation in the Cord Pilot Trial. Clinicians’ views and experiences are reported elsewhere [6].

## Methods

### Consent pathways in the Cord Pilot Trial

To help ensure that women were aware of the trial, information about the study was widely available in clinics and on wards at each hospital. Standard consent was the usual one-stage pathway for written consent. If birth was imminent (as the woman was in established labour or before emergency caesarean section) and thus there was insufficient time for this one-stage pathway, and the attending clinicians considered it appropriate, women were offered oral assent [4]. For oral assent, the woman was given a brief description of the trial and a short information leaflet (*see* Additional file 1: Appendix A). This process is in line with clinical governance advice for valid consent for research while in labour issued by the Royal College of Obstetricians and Gynaecologists [7]. After being given a brief summary of the study, with the opportunity to ask any questions, the woman was asked if she was willing to be recruited. If she said yes, she was randomised. If she did not give oral assent, she was not recruited. How long was required for oral assent depended on factors such as how much the woman already knew about the study and her knowledge and wishes about care during the third stage. After the woman gave birth, she was approached again so that the study could be explained in more detail, she could be given the long participant information sheet (*see* Additional file 1: Appendix B), any questions she had could be answered, and she could provide written consent for continued participation in follow-up.

Training in both pathways for consent was provided to site staff, including both research staff and clinicians. For the two-stage oral assent pathway, clinicians received training in offering oral assent because owing to time constraints, this could be undertaken only by the clinicians. The research staff received training in the second stage in this pathway of obtaining written consent. Training videos for obtaining consent in a range of clinical scenarios were provided to sites, and these included offering oral assent.

**Fig. 1:**
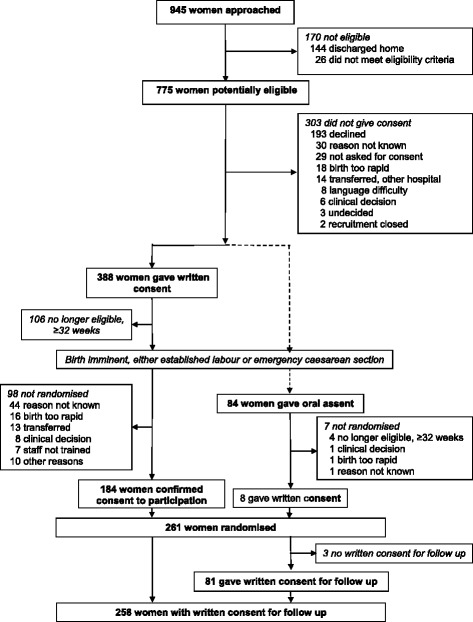
Participant flow with consent pathway

Of 945 women who were approached, 472 (50%) gave consent, and 193 (20%) declined participation. If women chose not to participate, they were informed that care would continue according to usual care at their hospital. The remaining women were discharged to home without having gone into labour, gave birth before reaching a decision, were transferred to another hospital or were not enrolled for other reasons. Oral assent was offered to 93 women, of whom 84 (90%) gave assent and 77 (83%) were randomised. Eight women who gave oral assent provided written informed consent before randomisation and so are considered within the group who were offered the usual one-stage consent pathway. In total, 261 women were randomised, of whom 69 women were randomised following oral assent alone (26% of women recruited to the trial). Figure 1 displays the participant flow in the study by consent pathway.

### Participants and procedure

This was a qualitative study using semi-structured interviews (*see* Additional file 2 for reporting guidelines for qualitative studies). A total of 179 women randomised to the Cord Pilot trial between April 2013 and October 2014 were eligible for this study. An invitation letter was sent by post to eligible women, along with a postage-paid envelope and reply slip for them to complete and return if they were willing to be interviewed. If there was no response after 2 weeks, a reminder letter was sent. Reminders were not sent to women whose baby had died. Women who responded were contacted, and an interview date was scheduled for a time convenient to them, to take place either at their home or by telephone. Interviews lasted approximately 30 minutes and were carried out by a female research assistant (CC) trained in qualitative methods. The interviewer would introduce herself and explain the purpose of the research. No one was present apart from the interviewer. The interview schedule (Additional file 1: Appendix C) consisted of open-ended questions to explore the women’s views and experiences of being offered participation in the Cord Pilot Trial. Probes were used to explore responses in more depth. Interviews were recorded and transcribed with all identifying information removed. Data collection ended when data saturation had been achieved, which was defined as when no new findings/themes were evident in the data. Information from the interviews was reviewed and discussed regularly between two authors (AS and CC), and when it was evident that no new themes were emerging, data collection ended.

### Data analysis

Inductive thematic analysis [8] was used to identify and analyse themes. Thematic analysis is a flexible research tool which can provide a rich and detailed account of the data. Compared with other, similar approaches (e.g., interpretative phenomenological analysis, grounded theory), thematic analysis is not theoretically bounded. Braun and Clarke’s [8] six phases of thematic analysis was followed: (1) familiarisation of the data, which involves the researchers immersing themselves in the data by reading and re-reading the data and noting any analytic observations; (2) coding, which involves generating labels for important features of the data which are generally relevant to the research question; (3) searching for themes, which involves looking across collected codes to identify similarity in the data; (4) reviewing themes, ensuring the themes correspond with both the coded extracts and the full dataset; (5) defining and naming themes, which involves the researchers conducting and writing a detailed analysis of each theme; and (6) write-up, which involves the researchers bringing together the analysis and data extracts to present a coherent reflection of the data. NVivo version 10 software (QSR International Pty Ltd., Doncaster, Australia) was used to manage codes and themes. For this report, direct quotes are coded (number = participant number; type of consent) to ensure anonymity.

## Results

Of the 179 women invited to participate, 27 (15%) responded and agreed to be interviewed. Three women could not be contacted to arrange an interview, and one later withdrew consent; hence, data for 23 (13%) women are reported here. Of these, 18 had been randomised following the usual written consent and 5 following oral assent with subsequent written consent. Women interviewed gave birth between 25^+2^ and 31^+6^ weeks of gestation at seven of the eight hospitals taking part in the trial. One woman had a twin pregnancy. The women were between 20 and 43 years old (mean 33 years, SD 6); most were married or cohabiting (Table 1); and almost half were educated to degree level. Two-thirds of the women had a caesarean birth. Most babies were home at the time of the interview, but one baby died shortly after birth, and another was still in the neonatal intensive care unit. Time between birth and the interview ranged from 1 to 14 months (mean 7.2 months). All but three women gave birth on the same day that they were randomised.

**Table 1:** Demographic information of the women and details about the birth and recruitment to the trial

Characteristics	*n* (%) (*n* = 23)
Highest level of education	
GCSEs/O levels	2 (9)
A levels/diploma/City & Guilds	7 (30)
Undergraduate degree	7 (30)
Postgraduate degree	4 (17)
Professional qualification	3 (13)
Marital status	
Married	11 (48)
Living with partner	9 (39)
Single	1 (4)
Other	2 (9)
Reason for preterm birth	
Antepartum haemorrhage	3 (13)
Pre-labour rupture	8 (35)
Spontaneous preterm labour	6 (26)
Pre-eclampsia	2 (9)
Placental abruption	1 (4)
Serious pre-existing health conditions^a^	3 (13)
Caesarean section	
Yes	14 (61)
Gestational age	
< 28 weeks	8 (35)
28–32 weeks	15 (65)
Consent pathway	
Standard written consent	18 (78)
Oral assent	5 (22)

*GCSE* General Certificate of Secondary Education

^a^Pre-existing conditions included severe asthma, Crohn’s disease and anti-phosphate lipid syndrome

Five major themes emerged as important factors in women’s experiences of the different consent pathways: (1) understanding of the implications of randomisation, (2) importance of staff offering participation, (3) information about the trial and time to consider participation, (4) trial secondary in women’s minds and (5) reasons for agreeing to take part in the trial.

### Understanding of the implications of randomisation

Not many women interviewed in this study showed a good understanding of randomisation and the reasoning for randomisation in trials. This was evident among women who were invited by formal written consent and had time to read the information sheet, consult with their partners and ask questions, as well as among the women who gave oral assent and had relatively less time to consider participation. Some women expressed a strong preference to be in the deferred cord clamping arm of the trial because they perceived delayed cord clamping to have more health benefits for their baby and therefore expressed disappointment that they were randomised to immediate clamping:

*‘But I was hoping I would have been randomised to delay and I wasn’t, so slightly disappointed at that. I felt disappointed at the time because I was thinking there are some benefits of it being delayed. I would have preferred to, because obviously that’s the research to see if there are benefits. So I was kind of thinking well there’s obviously some evidence enough to do a research trial with the public to see if there could be, so I was kind of thinking I would have preferred to have been there to see some of the benefits’. (2, written consent)*

One woman who had been allocated to immediate clamping (standard care) said, *‘I thought that my part wouldn’t help, because they didn’t do much, they just cut it straight away and he was breathing. So I thought, I’m not really involved am I’? (16, written consent).* This indicates a lack of understanding of RCTs, such as, in this case, the importance of a control group. One woman thought that she had ‘failed’ the trial because although she was allocated to the deferred clamping arm of the trial, the cord had to be cut before 2 minutes because it was too short:

*‘And then when I actually did give birth it was interesting because I felt like I’d really failed the trial. Because she couldn’t reach the bed so they had to cut the cord, they couldn’t keep her attached for the 2 minutes’. (14, oral assent)*

### Importance of staff offering participation

Staff members’ manner when they spoke to women about the trial was important to the women. All of the women interviewed were positive about the staff who approached them and offered consent. Some women, particularly those who gave oral assent when birth was imminent, mentioned the calm and professional attitude of the recruiting staff at a time of considerable anxiety. One woman describes how the doctor’s calm approach helped her focus on what was being said:

*‘He was very calm throughout the whole process, and I think his authority in the room allowed me to focus on what he was saying and to take on board what he was saying, rather than focusing on the anaesthetists and scanners and junior doctors. It was very much he talked to me directly, very clearly, concisely, didn’t mince his words, didn’t beat around the bush. Just very professional and very clear on what he was wanting and what was being said to me’. (1, oral assent)*

Many women also recalled that the staff were good at providing clear explanations about the trial, which was important because explanations were often taking place in a stressful context. Women also commented on the personable nature of the staff. For example, they were described as being warm, kind, friendly, and reassuring:

*‘He explained it, gave me information about it, which I think at the time would have been hard to take in except he was just so clear and very much like, this is what it is, don’t worry about, you don’t have to worry about it if you don’t want’. (22, written consent)*

*‘They were very personable, and they came to see me in person to collect my consent form. They didn’t put any pressure on me to consent, but it just meant that rather than me just being a number being entered into a trial they actually cared about me and wanted me to consider it carefully and have the opportunity to talk things through. So I thought that was good that you had that kind of personal interaction. Because like you said before it can be quite a stressful time going through pregnancy that’s going to have a preterm baby, so it was good that they did that’. (5, written consent)*

The staff’s friendly attitude was also mentioned by one woman (written consent) as one of the reasons she continued in the trial. Two women also mentioned that the research team were another source of support on the neonatal unit:

*‘Oh fine, [name] and the other girl whose name I can’t remember, they came to see us every day to talk about the trial. If we had any concerns, they were always there, always around. Very supportive not just about the trial, about everything we were going through. So I felt very confident and being part of the trial I suppose gave me an extra level of support in the unit because I had another friendly face to see and talk to’. (12, oral assent)*

Finally, the way staff presented the study to women was important in terms of making them feel comfortable to be able to say no to participation. This ‘no pressure’ approach was mentioned by 12 women (4 oral assent, 8 written consent) as one of the reasons they were happy to participate in the trial:

*‘Because I could have said no, there was no pressure. It was presented in a manner that even under the exceedingly stressful situation that we were in, it was presented in a manner that it was my decision, it was a yes or no. And if I had said no, they wouldn’t have pushed it on me’. (1, oral assent)*

Some women mentioned that *who* approached them was important. For example, one woman thought it was better that staff who were involved in their own or their baby’s care did not approach them about the study. This was because she would feel less pressured to say yes: ‘*If it was the obstetrician asking, I might say yes because it seems like there might be more benefits. But I think that it’s quite good that it’s the research team; I felt less pressured’ (2, written consent).* In contrast, another woman said that it was better *‘to get invited from the doctors that are going be involved. I think that’s probably the best way to do it’ (23, written consent).*

### Information about the trial and time to consider participation

All but one woman (oral assent) said they received sufficient information about the trial to make the decision to participate. However, when asked to elaborate on details of the information, only ten women reported receiving detailed information and being given sufficient time to consider participation before making a decision.

*‘Well, the research nurse talked to me, and then before the birth when I was in labour, the paediatrician talked to me in detail, and it was already really well explained by the nurse and in the documents what would happen.… You know, that’s the nice thing about me not going into labour straight away. They gave me the sheet and gave me as much time as I wanted to think about it, to read about it, and to, definitely. I think she came in the morning and she came back in the afternoon, something like that, so several hours to decide. I had decided in less than an hour I wanted to sign to do that’. (3, written consent)*

Of these women, one gave oral assent and nine gave written consent. In this instance, the woman who gave oral assent was approached and given information 1 week before going into labour but did not sign the consent forms until after birth, because she wanted to consult with her partner who was away. In contrast, five women described receiving only a brief summary of the trial, and of these, three women gave oral assent and two women received written consent.

*‘To be honest, that’s it. The conversation we had was all such a blur before we went into surgery. It was very much, “Would you like to be part of this trial?”’. (4, oral assent)*

*‘Not a great deal, to be honest. I haven’t really been told an awful lot; I don’t really know how long they did it for’. (6, written consent)*

However, women understood that this was more because of the nature of the situation rather than anyone’s fault. One woman who felt she did not have sufficient time describes that ‘*it was just the circumstance, not the nature of the doctors presenting it’ (1, oral assent).*

There were gaps in some women’s accounts regarding the potential benefits and risks of participation, uncertainty regarding which arm of the trial to which they were randomised, and what they would be asked to do for follow-up in the trial. Four women mentioned that they did not know how long would they would be followed and what they would have to do as part of this follow-up:

*‘I was clear about what would happen at delivery, but I wasn’t clear … there was a lot going, maybe I was told, but I can’t remember about sort of long term…. I filled a questionnaire in when I come home. But I don’t know what the long-term follow-up is as part of it. I probably was told, but I can’t remember any of that’. (2, written consent)*

Three women mentioned that they were not told which arm of the trial their baby was in and therefore did not know whether their baby received deferred or immediate cord clamping. Two of three of these women gave birth via caesarean section, one under general anaesthesia. All three stated that they had not asked at the time, but said that they would have liked to have been told. Another woman (oral assent), who was randomised to the deferred arm, described that her baby got cold and she wished that she had been told of this particular risk. One woman (written consent) misunderstood the purpose of the trial. She thought the trial was to do with care of the umbilical cord after birth.

### Trial secondary in women’s minds

Women were approached about the study and invited to participate at a time when they knew their baby was going to be born too early; for some, the birth was imminent. Unsurprisingly, therefore, the trial was a minor event in comparison to the birth of their baby. Twelve women discussed that they had lots of other things going on at the time and that once they agreed to participate in the trial, they did not give it much further thought (three oral assent, nine written consent).

*‘I think it was just that day, that’s how it was, really. I had other things going on; I didn’t know when I was going to be having a caesarean, so I kind of had other things on my mind probably than thinking about the trial. The trial was almost less of an importance for me’. (2, written consent)*

*‘I think there’s so much going on at that precise moment that it’s very hard to then categorise things and think, “Oh, actually, yes, I’ve signed up for that, that would be interesting”. I wasn’t even on that planet, you know. We were too busy thinking, “Oh my god, I’m going to have two babies in a minute, what are we going to do”? That sort of thing. I think once I’ve signed, I kind of boxed it, dismissed it and I was onto the next thing if that makes sense. Not in a ruthless way of not caring, but just we’d made our decision and that was it. We agreed to it, and off we went, I think’. (4, oral assent)*

### Reasons for agreeing to take part in the trial

Women discussed a range of reasons for agreeing to participate in the trial, the most common being that participation contributed to research:

*‘Basically, I think it’s like anything, isn’t it. Without research you don’t find out about things, so I totally support research. That was our feeling behind it, that, you know, if you don’t research these things, you don’t find out about it, do you? We’re completely open to research, and we think it’s a good thing, so it was important to take part’. (8, written consent)*

Many women (*n* = 13, 57%) also agreed to participate because they believed the trial posed no risk of harm to their baby and might give their baby a natural or better start, which was particularly important because they were going to be born very preterm.

*‘Well, I guess I just thought it sounded like a nice idea, and it made sense in terms of giving the baby the best start. I guess because we were looking at kind of having a premature baby that it seemed like that would be a good idea for her if that was possible’. (11, written consent)*

*‘Very easy decision, to be honest, because I knew there would be no danger to the baby to be left on the cord. I would have never if there was a risk, but I felt that there was no risk so there wasn’t any query of it really. It was easy as that’. (4, oral assent)*

Four women mentioned that one of the reasons they agreed to take part was that it was a simple trial and the intervention seemed ‘natural’:

*‘It’s just, I do think, like, just common sense that do something like this would make sure that the baby get that good blood supply with all the nutrient and all the goodness in it for little bit longer. So that’s why I said that because I think it feels very natural’. (20, written consent)*

Three women said they agreed to take part because of the possibility of seeing their baby being cared for beside them at the birth: *‘Because when we were talking about what was going to happen, it was definitely kind of one of the benefits, that the baby would be right beside you for a little bit longer’ (11, written consent).* Some women also mentioned that they were happy to take part because it seemed like a simple thing to do.

## Discussion

The aim of this study was to explore women’s views and experiences of two consent pathways for participation in a randomised trial of timing of cord clamping at very preterm birth: usual written consent or oral assent when birth was imminent. Women’s overall experiences of recruitment were similar, regardless of the consent pathway. Women valued clinicians’ positive manner when approaching them about the trial and felt they had appropriate information about the study. Although women recruited by oral assent had less detailed information at the time they gave assent, the women understood the time constraints and felt they were given sufficient information to make their decision about participation. Indeed, women who were recruited when birth was imminent and there was little time to make a decision felt it was easier to consider participation when clinicians provided minimal and clear information about the trial, especially because the trial was secondary in their minds at that time.

The main reason women gave for agreeing to participate in the trial was contributing to research that could help improve future care. Altruistic motivation is a commonly cited reason for participating in research [9, 10]. Other motivations were that the intervention posed little risk, seemed natural and might give the baby the best start. The view that deferring clamping of the umbilical cord is a more natural process might make recruitment by oral assent more likely to be acceptable. None of the women who gave oral assent mentioned or questioned that they had not been asked to sign a consent form, something which clinicians felt was an issue [6]. Some clinicians considered that because women did not have to sign anything at the point of oral assent there is not a clear record of the assent process. However, clinicians were asked to document in their notes that women had given oral assent. This was highlighted as being especially important if a participant was to have later questioned whether she did actually consent to participate. Some of the clinicians we interviewed also raised a concern that the standard written consent form may be off-putting to women because the form is ‘very legalistic’. However, none of the women raised this concern.

Some of the women discussed that it was important *who* approached them about participating in the trial. Opinion varied about whether it should be someone who was not involved in their care or someone who was looking after them and their baby. Some clinicians also thought that the person who approached women was important. Specifically, they thought the most appropriate person was one of the neonatal staff as part of their antenatal counselling to women and their families [6]. In this study, staff members’ ability to provide simple yet clear explanations about what the research entailed and their ‘no pressure’ approach were especially important to women who were approached when birth was imminent, given the stressful and time-critical context. These findings support literature that highlights the impact of clinical communication on potential participants’ decision making when deciding whether to enter a trial [11, 12]. This has implications for training in consent for intrapartum and perinatal trials or in other trials where consent is time-critical. Messages that provide support and reassurance and put medical content in everyday language affect how participants think and feel about their decisions.

According to the International Conference on Harmonisation good clinical practice guidelines, for consent to be valid, it must be properly informed and voluntarily given by a participant with the capacity to decide whether to take part in the research [13]. This is particularly relevant to obstetric and perinatal trials, such as the Cord Pilot Trial, where it is important to consider the context of women being in labour and/or in medical emergency situations which can involve high levels of stress and anxiety [14]. Whilst such situations do not mean that women lack the capacity to provide valid consent, they do mean that consent processes need to be adapted to suit this particular context. Studies have found that parents still want the opportunity to participate (or allow their infant to participate) in perinatal research and value their role as decision makers [15–17], and the oral assent pathway in our trial recognises that women about to give birth have the capacity to make the decision to participate. In our study, women recruited from both oral assent and written consent pathways showed understanding of the trial and intervention, demonstrating their ability to make decisions at a difficult time. Nevertheless, there was still some uncertainty for a few women over aspects of the trial, such as which intervention they were randomised to and follow-up. This uncertainty is consistent with other studies of participants’ understanding of trial procedures after the intervention, which have shown that parents often do not demonstrate a comprehensive understanding of randomisation and information they are given [18–20]. In our study, any misunderstandings could be due to the fact that the study involved mothers of babies who were going to be born very early and therefore the trial was secondary to other things in women’s minds. This further highlights the need for information to be minimal and easy to understand for women to be able to consider participation and give valid consent, whether written or oral.

### Strengths and limitations

To our knowledge, this is the first empirical study to evaluate a novel process of seeking consent for perinatal research when there is insufficient time for the usual single-stage informed consent process. Use of detailed qualitative methods allowed an in-depth exploration of women’s experiences of these two alternative consent pathways. Most women in our study were married or living with their partner and were well educated, which is not typical of women having very preterm birth. However, they may be more representative of women who agreed to participate in the trial because they have a similar distribution of age and gestation at birth. Our results are based on a single trial, and other factors may be more or less important in trials with different risk and benefit profiles. It is also possible that women may have been reluctant to criticise the trial because some of the research team were also involved in their baby’s care, but this is unlikely because the babies were now discharged and at home. Furthermore, it is possible that the stressful circumstances surrounding the birth influenced women’s recall of the consent process. For example, a previous study with parents of preterm babies demonstrated that just under half of the parents reported a blurred memory of the birth [21]. Finally, women who did not consent to take part in the trial were not interviewed, which means that we were unable to explore the experiences of this group.

## Conclusions

These findings have several implications for issues of intrapartum consent and future research on emergency neonatal interventions. Overall, women’s experiences of the two consent pathways were similar. Women in both pathways were positive about their experiences of being invited to participate in the trial, and they were particularly positive about the staff who offered consent. Although there were gaps in a minority of women’s accounts regarding the benefits and risks of the intervention, uncertainty over which arm of the trial they were randomised to and follow-up procedures, women were happy with the level of explanation received and expressed that they had sufficient time and information to make their decision to participate. Women recruited following oral assent understood that lack of time limited the information available to them. Oral assent therefore seems an acceptable alternative for offering consent in trials of low-risk interventions in emergency situations, and it has been incorporated into the latest guidelines for consent in obstetrics and gynaecology [7]. The use of oral assent therefore merits further use and evaluation. Future researchers might compare the two consent pathways in low-risk interventions to see how they impact parent (or patient) knowledge, satisfaction, anxiety, and views about consent.

## Additional files

Additional file 1: **Appendix A.** Short information leaflet – oral assent. **Appendix B.** Long information leaflet. **Appendix C.** Interview schedule. (DOCX 109 kb)

Additional file 2: Coreq checklist for qualitative studies. (DOCX 15 kb).
