# Supplementary material for: Women’s views and experiences of two alternative consent pathways for participation in a preterm intrapartum trial: a qualitative study
Source: Trials. 2017 Sep 9;18:422. doi: 10.1186/s13063-017-2149-3 (PMC5591564; doi:10.1186/s13063-017-2149-3)
Supplement: Supplementary file 1 — Appendix A. Short information leaflet – oral assent. Appendix B. Long information leaflet. Appendix C. Interview schedule. (DOCX 109 kb) [file 13063_2017_2149_MOESM1_ESM.docx]

**APPENDIX A: SHORT INFORMATION LEAFLET – ORAL ASSENT**

## [TO BE PRINTED ON TRUST HEADED PAPER]


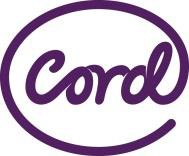


**Cord Pilot Trial**

**Parents’ Information Leaflet for Verbal Consent**

We are inviting you to take part in our research study because your doctors think you will give birth more than eight weeks before your due date (very preterm). Babies born at this time are very premature and need help with breathing, feeding and keeping warm. We want to try to improve the future care of mothers and babies born very premature.

## What is the purpose of the study?

Usually at the birth of a premature baby, the umbilical cord is clamped and cut straight away, the baby is placed in a plastic wrap to retain heat, and then cared for by the neonatologist (a doctor or nurse who is a specialist in the care of newborn babies) on a special table, which is usually at the side of the room. Clamping the cord stops blood flow between the placenta and the baby. If the cord is not clamped straight away, this blood flow may continue for several minutes, potentially transferring blood from the placenta to the baby. Waiting a few minutes before clamping the cord may help the baby adjust to life outside the womb. As we do not know when the best time is to clamp the cord for very preterm births, we are doing this study.

## What is the study?

For babies born very preterm, we want to compare (1) waiting for at least two minutes before cord clamping (deferred cord clamping) with (2) clamping the cord before 20 seconds. For deferred cord clamping, care for the baby will be provided at the bedside. If the cord is clamped before 20 seconds, the neonatal team will choose whether care for the baby is provided at the bedside or at the side of the room. In both cases the baby will receive the same care at birth, just in different places.

## Do I have to take part?

It is up to you to decide if you want to join the study or not. If you agree to take part, you are free to withdraw at any time, without giving a reason. If you choose not to take part, your care will continue in the normal way, and you and your clinician can decide when to clamp the cord.

## What will I have to do?

We are inviting you to give verbal consent now to take part in the study. If you agree to take part in the study, then at the birth you will have either ***(1) cord clamping after at least two minutes, or (2) cord clamping within 20 seconds***. If the cord is clamped after at least 2 minutes, care for your baby will be provided at your bedside. If the cord is clamped before 20 seconds, the neonatal team will choose whether care for your baby is provided at your bedside or at the side of the room. If you do take part, we will ask you to sign a consent form before you go home after the birth.

We will send you some short questionnaire to complete at various times after the birth, and we would like to find out how your child is doing at around two years of age.

## Who has reviewed the study?

This study has been reviewed and given favourable opinion by Nottingham 2 Research Ethics Committee (REC), the Nottingham University Hospitals NHS Trust, and the National Institute of Health Research Programme for Applied Research (which funded the study).

## Thank you for reading this information sheet

Pa

ge **1** of **1**


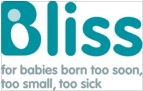


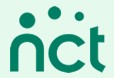


*PIS verbal consent Cord Pilot Trial version 1.0 22 April 2013*

**APPENDIX B: LONG INFORMATION LEAFLET**

# [TO BE PRINTED ON TRUST HEADED PAPER]


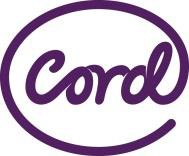


**Cord Pilot Trial Parents’ Information Leaflet**

*You are invited to take part in our research study. It is important you understand why the research is being done and what it would involve for you. Please take time to read this information. Talk to others if you wish, and ask if you would like more information.*

# What is the purpose of the study?

In the UK one in every 70 babies is born more than eight weeks before their due date (very preterm). These very premature babies have immature lungs, and often will need help with breathing, feeding and keeping warm. Usually at the birth of a premature baby, the umbilical cord is clamped and cut straight away, the baby is placed in a plastic wrap to retain heat, and then cared for by the neonatologist (a doctor or nurse who is a specialist in the care of newborn babies) on a special table, which is usually at the side of the room.

Clamping the cord stops blood flow between the placenta and the baby. If the cord is not clamped straight away, this blood flow may continue for several minutes, potentially transferring blood from the placenta to the baby.

Evidence from previous research suggests that waiting a few minutes before clamping the cord may help the baby to adjust to life outside the womb. However, this research was not of high enough quality, and the studies were too small, to give a clear answer. The research suggested that fewer babies needed blood transfusions for anaemia, and the risk of bleeding in the brain and the risk of a severe infection of the bowel were reduced. However, none of these babies have been contacted in childhood to find out how they are doing.

As we do not know when the best time is to clamp the cord for very preterm births, we are doing this study. For babies born very preterm, we want to compare waiting for at least two minutes before cord clamping (deferred cord clamping) with clamping the cord before 20 seconds. For deferred cord clamping, care for the baby will be provided at the bedside. If the cord is clamped before 20 seconds, the neonatal team will choose whether care for the baby is provided at the bedside or at the side of the room. In both cases the baby will receive the same care at birth, just in different places.

This study will help us to decide when the best time is to clamp the cord for very preterm births.

# Why have I been invited?

You have been invited to participate in this study as your doctors think you may give birth to your baby more than eight weeks before your due date.

# Do I have to take part?

It is for you to decide if you want to join the study or not. If you agree to take part, you are free to withdraw at any time, without giving a reason. This will not affect the standard of care you and your baby receive. If you choose not to take part, your care will continue in the normal way, and you and your clinician can decide when to clamp the cord.

**What will I have to do?**

You will be asked to sign a consent form before you participate in the study. If the birth happens quickly and there is not enough time for you to sign the form, you may be asked to give verbal consent. If you give verbal consent, then before you leave hospital you will be asked to sign a consent form to allow us to access your hospital records, and to contact you later to find out how you and your baby are.

If you take part in the study, then at the birth you will have either (1) cord clamping after at least two minutes, or (2) cord clamping before 20 seconds. The decision about which you receive will be decided by chance (rather like tossing a coin) and neither you nor your doctor will be able to choose. This is important, as it ensures a fair test between the two different forms of care. If the cord is clamped after at least 2 minutes, care for your baby will be provided at your bedside. If the cord is clamped before 20 seconds, the neonatal team will choose whether care for your baby is provided at your bedside or at the side of the room.

We will ask you to complete a short questionnaire two – three months after the birth, and another at one year after the birth. These will usually be posted to you at home, with a Freepost return envelope, but if your baby is in hospital at six weeks you may be given the questionnaire there instead. Completing each questionnaire takes about 15 minutes.

We will write to you again when your child is around two years of age to find out how your child is doing. We will post you a questionnaire which will take around 10 – 15 minutes to complete. The questionnaire will be posted to you at your home, with a Freepost return envelope. We may contact you again in the future to find out how your child is doing.

To help us stay in touch with you and your child and to find out how you are, we will ask your permission to register you and your child with the NHS Information Centre.

# What are the possible disadvantages and risks of taking part?

In the past, a disadvantage of deferring cord clamping for very preterm births was thought to be that it would delay the neonatologist providing care for the baby. But the neonatologists are now able to provide care for the baby at the mother’s bedside. Therefore care for these babies will be the same, regardless of when the cord is clamped.

# What are the possible benefits of taking part?

We cannot promise the study will help you or your baby but the information we get from this study will help to improve the future care of mothers and babies born very premature.

# What happens when the research study stops?

We will write to you at the end of the study, and explain that we will send you a summary of the study results unless you ask us not to. If you decide not to take part in the follow up assessment, we will use the data collected up to that point, unless you ask us not to.

If the evidence shows that there is an important difference between the two forms of care, then we may want to contact you again in the future to find out how your child is.

# Will my taking part in this study be kept confidential?

Yes. All information we collect about you will be kept in the strictest confidence. In order to be able to contact you about your own and your baby’s health, your name and contact details will be made available to the researchers running this study and held at the co- ordinating centre in Nottingham, and not just held by your local study doctor. These detaiwill be kept securely, with access restricted. You will not be named or otherwise identified in any study publication.

# Involvement of your General Practitioner (GP)

We will write to your GP to explain that you are taking part in the study. We may contact your GP if we lose contact with you, for instance because you move, unless you ask us not to.

# What will happen to the results of the research study?

The findings from this initial (pilot) study will be used to plan the larger study. They will also be presented at medical meetings and submitted for publication in medical journals and newsletters. We will send you updates on the research and a summary of the study results unless you ask us not to.

# What if there is a problem, or I want independent advice?

If you have a concern or questions about any aspect of this study, you should ask to speak to the local researchers (their contact details are at the end of this leaflet). You can also contact the Trial Co-ordinating Centre at the Nottingham Clinical Trials Unit.

If you want to talk to someone else you can contact the hospital’s Patient Advisory and Liaison Service (PALS). They will be able to give you advice about how to contact someone for independent advice. Ask one of the doctors or nurses how to contact them.

If you wish to make a complaint, or have any concerns about any aspect of your care during the course of the study, the normal NHS complaints service is available to you. If you are harmed by taking part in this study, there are no special compensation arrangements. If you are harmed due to someone’s negligence, then you may have grounds for legal action but you may have to pay for this

# Who has reviewed the study?

This study has been reviewed and given favourable opinion by Nottingham 2 Research Ethics Committee (REC). The REC looks after the rights, well being and dignity of people invited to take part in research studies. The study has also been reviewed by the Nottingham University Hospitals NHS Trust, and the National Institute of Health Research Programme for Applied Research (which has funded the study).

# Further information and contact details

Further information is available on

[www.nottingham.ac.uk/nctu/trials/pregnancy-and-childbirth.aspx](http://www.nottingham.ac.uk/nctu/trials/pregnancy-and-childbirth.aspx)

***Contact details of your local research team***

Name:

Telephone:

*Alternatively, contact the study team at the Nottingham Clinical Trials Unit:*

Angela Pushpa-Rajah, Cord Pilot Trial

Nottingham Clinical Trials Unit, Nottingham Health Science Partners C Floor, South Block, Queens Medical Centre

Nottingham NG7 2UH

Tel: 0115 8844936 Email: [angela.pushpa-rajah@nottingham.ac.uk](mailto:angela.pushpa-rajah@nottingham.ac.uk)

Pag

e **3** of **3**

*PIS Cord Pilo*


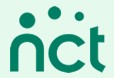


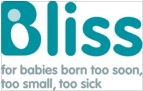


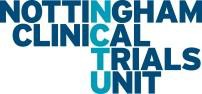


**Thank you for reading this information sheet**

*t Trial Final version 3 dated 29 January 2014*

**APPENDIX C: INTERVIEW SCHEDULE**

**Opening questions**

1. Please can you describe what led up to your baby’s birth?
2. What can you remember about being in the trial?

**Experience of the recruitment process**

- Please can you tell me what happened when you were approached about going into the trial?
  - What can you remember being told or reading about the trial?
  - Had you heard of the trial before?
  - Were you clear about what being in the trial would mean for you and your baby?
- Can you tell me about what happened when you agreed to take part in the trial?
  - How did it feel at the time?
  - To what extent did you feel it was your decision?
  - Did you feel, at that time, that you were able to make that decision?
    1. Did you have enough information?
    2. Did you have enough time to make the decision?
  - Did you feel that you could say no if you wanted to?
  - Did you feel you could change your mind if you wanted to?
  - What were your reasons for agreeing to take part in the trial?
- Were you clear about what was going to happen to you and your baby/had happened to you and your baby as part of the Cord Pilot Trial?
- **[for women who gave oral assent]** How did you feel about getting some information about the trial before the birth, and then having more information and time to discuss the trial after your baby was born?
- Was there anything about being invited into the trial and agreeing to take part that you felt particularly comfortable with or liked?
- Was there anything about being invited into the trial and agreeing to take part that you were particularly unhappy with, or disliked?
- Was there anything that could have been done differently to improve the way you were invited into the trial?

**Additional Question**

- Have you been involved with/participated in any other research trials?
